# Public awareness of and opinions on the use of mathematical transmission modelling to inform public health policy in the United Kingdom

**DOI:** 10.1098/rsif.2023.0456

**Published:** 2023-12-20

**Authors:** Ruth McCabe, Christl A. Donnelly

**Affiliations:** ^1^ Department of Statistics, University of Oxford, Oxford, UK; ^2^ Pandemic Sciences Institute, University of Oxford, Oxford, UK; ^3^ NIHR Health Protection Research Unit in Emerging and Zoonotic Infections, University of Liverpool, Liverpool, UK; ^4^ MRC Centre for Global Infectious Disease Analysis, Imperial College London, London, UK

**Keywords:** COVID-19, survey, public opinion, sampling, UK

## Abstract

Mathematical modelling is used to inform public health policy, particularly so during the COVID-19 pandemic. As the public are key stakeholders, understanding the public perceptions of these tools is vital. To complement our previous study on the science-policy interface, novel survey data were collected via an online panel (‘representative’ sample) and social media (‘non-probability’ sample). Many questions were asked twice, in reference to the period ‘prior to’ (retrospectively) and ‘during’ the COVID-19 pandemic. Respondents reported being increasingly aware of modelling in informing policy during the pandemic, with higher levels of awareness among social media respondents. Modelling informing policy was perceived as more reliable during the pandemic than in reference to the pre-pandemic period in both samples. Trust in government public health advice remained high within both samples but was lower during the pandemic in comparison with the (retrospective) pre-pandemic period. The decay in trust was greater among social media respondents. Many respondents explicitly made the distinction that their trust was reserved for ‘scientists’ and not ‘politicians’. Almost all respondents believed governments have responsibility for communicating modelling to the public. These results provide a reminder of the skewed conclusions that could be drawn from non-representative samples.

## Highlights

— Awareness of modelling in informing policy increased during the pandemic.
— Trust in government public health advice remained high over time.
— Respondents felt communication was the responsibility of the government.
— Respondents were aware of modelling primarily through the news and/or social media.
— Conclusions based on non-representative samples may be skewed.

## 1. Introduction

Scientific advisory mechanisms in the United Kingdom of Great Britain and Northern Ireland (UK) were brought into the full view of the public eye during the COVID-19 pandemic. Restrictive physical distancing measures, such as lockdowns, affected all within society and, rightfully, sparked a widespread interest in the evidence underpinning such decision-making by the government. As society reflects on the events of the COVID-19 pandemic, particularly in anticipation of the outcome of the public inquiry into the UK government's handling of the pandemic [1], the use, or not, of scientific evidence to inform policy will be examined closely.

Mathematical transmission modelling is a key component of scientific evidence presented to the government during outbreaks of infectious diseases: the COVID-19 pandemic was no exception [2,3]. Already, there is a growing body of work focusing on the use of this set of tools during the recent pandemic [4–6], including one by the authors of this study [2]. In our previously published study, we examined the interplay between mathematical transmission modelling, scientists, the media and the public throughout the first year of the pandemic [2]. Participants in our research, namely scientific advisors and public communication experts, indicated that direct communication with the public had been challenging. As the success of public health policy often depends upon the cooperation of the public, it is important that the public perception of scientific advice, in this case specifically mathematical modelling, is understood. Much of the research into public opinions throughout the COVID-19 pandemic has related to vaccine hesitancy [7–9] or adherence to public health and social measures [10–13]. Although Marshall *et al*. [14] interviewed members of the public regarding Test, Trace and Isolate policies with a view to improving assumptions underpinning mathematical modelling, respondents were not explicitly asked about their perceptions of modelling.

We sought to complement our initial study [2] by gathering and analysing data on the public's awareness of and opinions on the use of mathematical transmission modelling to inform public health policy in the UK via an online questionnaire covering topics such as means of awareness and responsibility of the communication of modelling, feelings of reliability of modelling in informing policy, and trust in government public health policy (table 1). Even with sufficient financial resources, obtaining a ‘representative’ sample of the population is notoriously difficult and thus the interpretation of the resulting data must be cautious regarding the opinions of the adult population in the UK. Consequently, we collected data via two sampling methods: via an online panel (using the platform *Prolific Academic* [15] and considered a ‘representative’ sample in the context of this paper) and via social media (using *Twitter* [16] and considered a ‘non-probability’ sample). As such, the goals of this study are twofold: to better understand public awareness of and opinions on the use of modelling to inform public health policy but also, critically, to explore and interpret any systematic differences between the two samples obtained (online panel and social media).

**Table 1.** Questions, and multiple-choice answers where relevant, for the survey to the public. The two compulsory questions are highlighted with an asterisk (*). Totals and percentages are based on responses with ‘complete’ demographic data, namely age group, gender, sector and vaccination status within each sample.

question	question type	response options (if multiple-choice)	number of online panel respondents (*n* (%))	number of social media respondents (*n* (%))
**General**				
How would you describe a transmission model?*	Open-ended		504	202
What level of confidence do you have in your description?*	Multiple-choice	Not at all confident	200 (40%)	70 (35%)
		Moderately confident	262 (52%)	111 (55%)
		Very confident	42 (8%)	21 (10%)
**Before the COVID-19 pandemic**				
*For the purposes of this research, a transmission model is defined as a mathematical model of the spread of a virus through a population in order to simulate quantities such as, but not limited to, the daily number of deaths, new infections and hospitalisations due to the virus.*				
Prior to the COVID-19 pandemic, how were you aware of transmission modelling? Please select all that apply.	Multiple-choice	I was not aware	314 (62%)	74 (37%)
		Newspaper (online or print)	69 (14%)	34 (17%)
		News show (TV or online)	68 (13%)	27 (13%)
		Social media	34 (7%)	16 (8%)
		Internet search	49 (10%)	22 (11%)
		Academic reports and papers	53 (11%)	48 (24%)
		Other	36 (7%)	45 (22%)
		Did not answer question	0 (0%)	0 (0%)
Before the COVID-19 pandemic, where do you think those who developed and used transmission models work?	Open-ended			
Prior to the COVID-19 pandemic, were you aware of the use of transmission models in informing public health policy (eg. the model of the spread of pandemic influenza)?	Multiple-choice	Yes	137 (27%)	116 (57%)
		No	274 (54%)	49 (24%)
		Unsure	91 (18%)	37 (18%)
		Did not answer question	2 (0%)	0 (0%)
Prior to the COVID-19 pandemic, on a scale of 1–10 with 1 being ‘extremely unreliable’ and 10 being ‘extremely reliable’ how did you feel about the use of transmission models in informing public health policy? Please select a number below.	Multiple-choice	1	11 (2%)	4 (2%)
		2	3 (1%)	0 (0%)
		3	23 (5%)	13 (6%)
		4	24 (5%)	5 (2%)
		5	112 (22%)	47 (23%)
		6	60 (12%)	27 (13%)
		7	118 (23%)	35 (17%)
		8	110 (22%)	47 (23%)
		9	28 (6%)	19 (9%)
		10	11 (2%)	4 (2%)
		Did not answer question	4 (1%)	1 (0%)
Prior to the COVID-19 pandemic, how much did you trust government advice regarding public health issues?	Multiple-choice	High level of trust	131 (26%)	65 (32%)
		Moderate level of trust	322 (64%)	117 (58%)
		No trust whatsoever	51 (10%)	20 (10%)
		Did not answer question	0 (0%)	0 (0%)
Please provide a brief explanation regarding your answer to the previous question.	Open-ended			
**During the COVID-19 pandemic**				
*For the purposes of this research, a transmission model is defined as a mathematical model of the spread of a virus through a population in order to simulate quantities such as, but not limited to, the daily number of deaths, new infections and hospitalisations due to the virus.*				
During the COVID-19 pandemic, how were you aware of transmission modelling? Please select all that apply.	Multiple-choice	I was not aware	114 (23%)	6 (3%)
		Newspaper (online or print)	204 (40%)	123 (61%)
		News show (TV or online)	300 (60%)	142 (70%)
		Social media	147 (29%)	125 (62%)
		Internet search	122 (24%)	72 (36%)
		Academic reports and papers	86 (17%)	97 (48%)
		Other	19 (4%)	23 (11%)
		Did not answer question	0 (0%)	0 (0%)
During the COVID-19 pandemic, where do you think those who developed and used transmission models work?	Open-ended			
During the COVID-19 pandemic, have you been aware of the use of transmission models in informing public health policy (e.g. the implementation of so-called ‘lockdown’ measures)?	Multiple-choice	Yes	374 (74%)	195 (97%)
		No	75 (15%)	2 (1%)
		Unsure	47 (9%)	4 (2%)
		Did not answer question	8 (2%)	1 (0%)
During the COVID-19 pandemic, on a scale of 1–10 with 1 being ‘extremely unreliable’ and 10 being ‘extremely reliable’ how did you feel about the use of transmission models in informing public health policy? Please select a number below.	Multiple-choice	1	11 (2%)	10 (5%)
		2	9 (2%)	6 (3%)
		3	13 (3%)	9 (4%)
		4	21 (4%)	5 (2%)
		5	62 (12%)	24 (12%)
		6	56 (11%)	20 (10%)
		7	105 (21%)	36 (18%)
		8	129 (26%)	53 (26%)
		9	68 (13%)	30 (15%)
		10	26 (5%)	9 (4%)
		Did not answer question	4 (1%)	0 (0%)
During the COVID-19 pandemic, how much did you trust government advice regarding public health issues?	Multiple-choice	High level of trust	88 (17%)	18 (9%)
		Moderate level of trust	291 (58%)	108 (53%)
		No trust whatsoever	124 (25%)	76 (38%)
		Did not answer question	1 (0%)	0 (0%)
Please provide a brief explanation regarding your answer to the previous question.	Open-ended			
How much do you know about how transmission modelling has been used throughout the COVID-19 pandemic?	Multiple-choice	Too little	233 (46%)	60 (30%)
		About right	257 (51%)	122 (60%)
		Too much	10 (2%)	17 (8%)
		Did not answer question	4 (1%)	3 (1%)
Who do you think has the responsibility of ensuring that the public are informed about the use of modelling in policy decisions, particularly in the COVID-19 pandemic? Please select all that apply.	Multiple-choice	The government	462 (92%)	183 (91%)
		Those who develop transmission models	198 (39%)	130 (64%)
		Those who use transmission models	217 (43%)	129 (64%)
		The media	221 (44%)	121 (60%)
		Myself, as a member of the public	108 (21%)	68 (34%)
		None of the above	1 (0%)	2 (1%)
		Did not answer question	1 (0%)	0 (0%)
If answered ‘None of the above’, please write who you think has the responsibility of ensuring that the public are informed about the use of modelling in policy decisions here.	Open-ended			
How do you feel when government advice changes based on new scientific evidence?	Multiple-choice	I have more trust in the advice.	221 (44%)	111 (56%)
		I have less trust in the advice.	67 (13%)	19 (10%)
		My level of trust remains unchanged.	214 (42%)	70 (35%)
		Did not answer question	2 (0%)	2 (1%)
Please provide a brief explanation regarding your answer to the previous question.	Open-ended			
Please provide any further comments about any of the previous questions.	Open-ended			
**Demographic information**				
*Please note that you will not be able to be identified based on any information you provide in this section.*				
Please select your age group.	Multiple-choice	18–25	71 (14%)	4 (2%)
		26–35	90 (18%)	10 (5%)
		36–45	92 (18%)	23 (11%)
		46–55	87 (17%)	58 (29%)
		56–65	125 (25%)	81 (40%)
		66+	39 (8%)	26 (13%)
Please select your gender.	Multiple-choice	Female	260 (52%)	104 (51%)
		Male	240 (48%)	95 (47%)
		Non-binary	1 (0%)	0 (0%)
		Prefer not to say	3 (1%)	3 (1%)
Please select the option below which most closely matches the sector you work in.	Multiple-choice	Agriculture	1 (0%)	0 (0%)
		Business, finance and technology	75 (15%)	29 (14%)
		Creative	21 (4%)	5 (2%)
		Education	68 (13%)	51 (25%)
		Health and social work	69 (14%)	30 (15%)
		Production (energy, manufacturing, mining)	30 (6%)	12 (6%)
		Public	36 (7%)	16 (8%)
		Research	17 (3%)	21 (10%)
		Retail, hospitality and tourism	48 (10%)	4 (2%)
		Other	139 (28%)	34 (17%)
**COVID-19 vaccination**				
Have you been vaccinated for COVID-19?	Multiple-choice	Yes	439 (87%)	195 (97%)
		No, but I plan to in the future	41 (8%)	1 (0%)
		No, and I do not plan to	24 (5%)	6 (3%)

## 2. Material and methods

### 2.1. Survey design and distribution

This study has approval from the Medical Sciences Interdivisional Research Ethics Committee at the University of Oxford with Ethics Approval Reference R76166/RE001. The Participant Information Sheet (PIS), provided in the electronic supplementary material, sets out the instructions given to participants ahead of taking the survey.

We designed an online survey of 24 questions, comprising 16 multiple-choice and 8 open-ended questions (table 1). The survey was split into four sections of unequal length.

To begin, participants were asked to describe a transmission model and state the level of confidence that they have in their description. These were the only two compulsory questions in the survey. Once participants answered this question, a description of transmission models in the context of this survey was given so that answers given in the rest of the survey were relevant to the research questions at hand.

The middle two sections contained most questions which form the basis of the analysis in this paper. Participants were asked a series of questions about their awareness of and opinions on transmission modelling to inform policy before the COVID-19 pandemic. The next section then repeated these questions, also alongside some additional questions, but asked participants to answer in relation to the period during the COVID-19 pandemic. As questions about time periods were both responded to simultaneously (summer 2021), it is important to be aware of possible recall bias of participants in reference to the period prior to the pandemic.

Finally, participants were asked to provide demographic information, namely age group, gender, the sector that they work in and their COVID-19 vaccination status.

The survey was designed in Microsoft Forms. The questions are presented in full in table 1.

Participants were gathered via two methods: via an online panel and via social media. Only individuals aged over 18 and residing in the UK were permitted to partake in the survey.

The online panel was surveyed via *Prolific Academic*, an online platform with a large, diverse database of individuals who have consented to participate in research on a multitude of different topics. Recruitment to Prolific Academic primarily relies on ‘word of mouth’ [17]. The company actively advertises that ‘we currently offer representative samples of two national populations: the United Kingdom and the United States' [18], but also note that ‘no sample can ever be 100% representative, so using a representative sample does not guarantee that your results will be perfectly generalizable to the population (although it does help to improve generalizability)’ [18]. Our survey was sent to a sample of approximately 500 people signed up to the platform, who were selected to target ‘representation of the UK population’ across a range of demographic variables, including ‘Age’, ‘Sex’, ‘Country of Birth’ and ‘Employment Status’ [19]. As such, this sample is considered a ‘representative’ sample within the context of this paper. Responses were gathered across 7–9 July 2021.

Alongside this, we shared our survey on the social media platform *Twitter* by tweeting a link to it, and asked collaborators and accounts associated with the departments to which the authors are affiliated to also do so. An overview of the accounts that tweeted or quote tweeted the survey is presented in electronic supplementary material, table S1. Undoubtedly, a network effect will have influenced who saw the survey in their timeline and who will have selected to participate. Consequently, this sample is considered a non-probability sample. *A priori*, it was expected that this method of participant recruitment could substantially skew the demographics and answers of the participants within this sample. The survey was first shared on Twitter on 7 July 2021. Although responses were received until 3 September 2021, the vast majority were obtained between 7 and 20 July 2021.

### 2.2. Statistical analysis

Data were filtered so as to only include observations with all four demographic variables answered (age group; gender; sector; vaccination status). Throughout, percentages of respondents have been rounded to the nearest integer.

#### 2.2.1. Classification of open-ended responses

Responses to three open-ended questions, namely ‘How would you describe a transmission model?’; ‘Before the COVID-19 pandemic, where do you think those who developed and used transmission models worked?’ and ‘During the COVID-19, where do you think those who developed and used transmission models worked?’, were categorized to aid the interpretation of the results. Categories were not predetermined and were refined having considered the entirety of the data.

Descriptions of transmission modelling were deemed to be more relevant to the context of our research in relation to infectious disease epidemiology and/or to mathematics and statistics more broadly. Each response was considered individually to assess the sentiment of the description. For example, the use of words such as ‘virus’, ‘disease’, ‘infection’, ‘illness’, ‘epidemic’ or those akin to ‘COVID-19’ (e.g. ‘coronavirus’, ‘covid’), or others such as ‘statistics’, ‘mathematical’, ‘data’, ‘prediction’ or ‘simulation’, were all interpreted as capturing the essence of ‘disease transmission and control modelling’ and thus were considered as ‘more relevant’, compared with ‘less relevant’, to the topic at hand.

Answers to the open-ended question about where transmission modellers work (prior to and during the COVID-19 pandemic) were also assessed on an individual basis. If respondents stated multiple places of work, each were categorized individually. While ‘academia’ and references to ‘university’ were classified as ‘Research (Academia)’, any other reference to ‘research’ or ‘science’ or ‘laboratories’ were classed as ‘Research (Unspecified affiliation)’. ‘Public health bodies' encompassed either direct reference to the term ‘public health’ or specific organizations such as the World Health Organization or Public Health England (the United Kingdom Health Security Agency now combines elements of Public Health England and NHS Test and Trace [20]). Responses were classified as ‘Government’ if either that word was used, a specific department was named, such as the ‘Department of Health’ or reference was made to the ‘civil service’. Any reference to ‘journalists’ or ‘the press’ or ‘the media’ were clustered together under the heading ‘Media’, but not those stating ‘Social media’ (of which there were extremely few). ‘Healthcare services’ encompassed reference to ‘health bodies’ without direct mention of ‘public health’. For example, this includes terms such as ‘hospitals’, ‘the NHS’ and ‘healthcare providers'. References of ‘advisory’ to government, or ‘advisers’, including the use of ‘SAGE’, was classified as ‘Advisory roles’. All other responses were placed into ‘Other’. In most instances, answers were classified in more than one category as they were not mutually exclusive.

Responses to the remaining open-ended questions were not explicitly categorized but, as above, were considered on an individual basis.

### 2.3. Analysis of answers to multiple-choice questions

Chi-squared tests were used to test for differences in the proportions of respondents selecting specific answers to multiple-choice questions across samples. Within respondents, Wilcoxon signed rank tests were used to assess the differences between time periods within respondents. Questions in which there was a discrete scale from one extreme to the other, for example: ‘Not at all confident’ to ‘Very confident’; or ‘No trust whatsoever’ to ‘High level of trust’, were enumerated on a scale where 0 represented the middle strength option (e.g. ‘Moderately confident’ or ‘Moderate level of trust’), with 1 assigned to the highest level (e.g. ‘Very confident’ or ‘High level of trust’) and −1 assigned to the lowest level (e.g. ‘Not at all confident’ or ‘No trust whatsoever’) for each time point. Cases with a binary outcome, for example, whether or not a respondent selected a specific means of being aware of transmission modelling (e.g. ‘Newspaper (online or print)’), were enumerated as 1 if this option was selected and 0 otherwise. Specific details for each question, alongside results tables, are presented in the electronic supplementary material.

#### 2.3.1. Reliability scores

Answers to the question ‘On a scale of 1–10 with 1 being ‘extremely unreliable’ and 10 being ‘extremely reliable’, how do you feel about the use of transmission models in informing public health policy’?, both ‘Prior to the COVID-19 pandemic’ and ‘During the COVID-19 pandemic’ are referred to as ‘reliability scores’ throughout. This is the only question in which participants were asked for a numeric answer. Mann–Whitney U tests were used to assess differences in scores between samples within time periods, while Wilcoxon signed rank tests were used to assess differences in scores between time periods within respondents.

#### 2.3.2. Regression analysis

Linear regression was used throughout the study to quantify the relationships between the responses to each multiple-choice question (outcome) and other variables (explanatory, primarily demographic variables, but also occasionally answers to other multiple-choice questions). For example, we regressed responses to the question ‘Were you aware of the use of transmission models in informing public health policy?’, with the answer scale enumerated as described above, on age and sex (electronic supplementary material, table S3). Due to the number of multiple-choice questions, many linear regression models were analysed with the specific details and results presented in full in the electronic supplementary material.

Throughout, identical regression models were fitted separately on data from the online panel and social media samples. Chi-squared tests were used to assess the overall significance of categorical variables in each model. In the main text, results of these tests are reported if they show statistical significance at the 0.01 level.

When assessing the relationship between demographic and other variables, due to the large number of employment categories and limited number of respondents selecting that they are unvaccinated, only age group and gender were included within regression models. The youngest age group, 18–25 years, and females, the most frequently reported gender, were designated as the reference levels.

## 3. Results

Participants totalled 509 and 207 across the online panel and social media samples, respectively (total participants 716). Of these, 504 (99%) and 202 (98%) had ‘complete’ demographic data and thus were included in the analysis (total participants 706). Table 1 includes the results of the multiple-choice questions by sample. Response rates to multiple-choice question were high in both samples, with at least 98% of respondents with ‘complete’ demographic data answering each question.

Aside from the demographics of the respondents, the results are discussed with respect to four main themes: awareness, reliability, communication, and trust in government advice. Extensive further analyses are presented in the electronic supplementary material.

### 3.1. Respondent demographics

**Figure 1.**
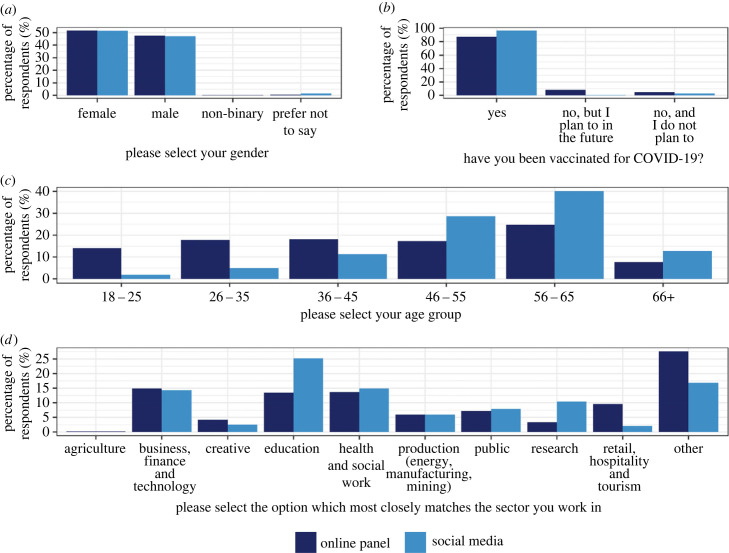
Demographics of survey participants stratified by sample. (*a*) Gender. (*b*) COVID-19 vaccination status. (*c*) Age group. (*d*) Sector participants work in. Underlying data are presented in table 1.

Gender had similar distributions across samples (table 1; figure 1*a*), with females holding a slight majority (52% and 51% for the online panel and social media, respectively).

There was heterogeneity across the samples in terms of age group and employment sector (table 1; figure 1*c,d*). Age groups of the online panel respondents were approximately evenly distributed except for the oldest age group (66+ years) in which there were substantially fewer participants. By contrast, the number in each age group of the social media sample steadily increased up to 56–65 years, which had a notably larger percentage of participants than other groups (40%), before also falling in the 66+ category. While half of the employment sector options had fairly consistent percentages of participants working in them across samples, (namely: ‘Agriculture’, ‘Business, finance and technology’; ‘Health and social work’; ‘Production (energy, manufacturing, mining)’; ‘Public’), there were fairly large differences elsewhere. For example, social media respondents were significantly more likely than their online panel counterparts to work in ‘Education’ (chi-squared test: $\chi_{df=1}^{2}=13.39;\;n=706;\;p\text{-value}<0.001$) or ‘Research’ (chi-squared test: $\chi_{df=1}^{2}=12.62;\;n=706;\;p\text{-value}<0.001$). This is likely to be attributable to the fact that it was predominantly researchers or research institutions sharing the survey (electronic supplementary material, table S1).

Social media respondents were also significantly more likely to be vaccinated against COVID-19 than online panel respondents (97% and 87%, respectively; figure 1*b*; chi-squared test: $\chi_{df=1}^{2}=13.00;n=706;\;p\text{-value}<0.001$). However, similar percentage of participants in both samples recording having no intention of being vaccinated (5% and 3%, respectively; chi-squared test: $\chi_{df=1}^{2}=0.74;\;n=706;$ p-value=0.390), with the remaining differences explained by those indicating their intention to be vaccinated in the future.

### 3.2. Awareness of mathematical transmission modelling

Within samples and time periods, levels of awareness of mathematical transmission modelling (herein referred to as ‘modelling’) generally were indistinguishable from the levels of awareness of modelling being used in policy specifically (electronic supplementary material, table S2). An exception was online panel respondents reporting being significantly less aware of modelling being used for policy than modelling in general in reference to the pre-pandemic period (Wilcoxon signed rank test: p-value<0.001).

**Figure 2.**
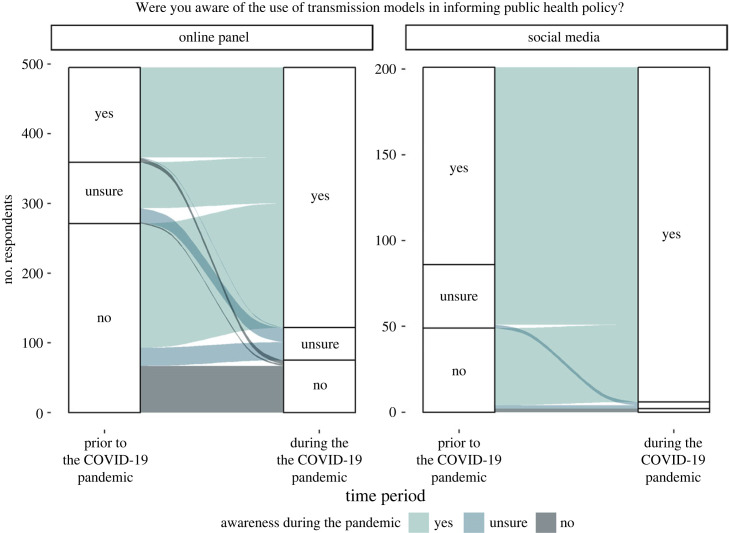
Awareness of modelling informing public health policy. Responses to the question ‘Were you aware of the use of transmission models in informing public health policy?’ both ‘Prior to’ (reported retrospectively) and ‘During’ the COVID-19 pandemic for the online panel and social media samples. Observations corresponding to a respondent who did not answer the question for at least one time point were removed from the figure to aid visual presentation (*n* = 8 online panel; *n* = 1 social media). Due to the vast majority (97%) of social media respondents reporting being aware of transmission modelling during the COVID-19 pandemic, the options of ‘Unsure’ and ‘No’ are unable to be labelled but appear in the same order as in the three preceding response pillars with ‘Unsure’ above ‘No’. Underlying data are presented in table 1.

The majority of participants across both samples were aware of modelling being used to inform public health policy in the period of the pandemic (table 1; figure 2), with these levels being significantly higher than those reported for the pre-pandemic period (Online panel: 27% pre-pandemic, 74% during; Wilcoxon signed rank test  p-value<0.001; Social media: 57% pre-pandemic, 97% during; Wilcoxon signed rank test  p-value<0.001). However, social media respondents were significantly more likely to be aware of modelling informing policy than online panel respondents regarding each time period (chi-squared test: prior: $\chi_{df=1}^{2}=56.05;\;n=706;\;p\text{-value}<0.001$; during: $\chi_{df=1}^{2}=44.55;\;\;n=706;\;\;p\text{-value}<0.001$).

Online panel respondents were primarily aware of modelling through ‘News show (TV or online)’ or ‘Newspapers (online or print)’ across both time periods (electronic supplementary material, figure S1; table S4). However, social media respondents were primarily aware of modelling through ‘Academic reports and papers' prior to the COVID-19 pandemic, but ‘News show (TV or online)’, ‘Newspapers (online or print)’ and ‘Social media’ were the most popular selections during the pandemic. For both time periods, social media respondents were significantly more likely than the online panel to select ‘Academic reports and papers' (electronic supplementary material, table S5; chi-squared test: prior: p-value<0.001; during:  p-value<0.001), which could be driven in part by the significantly greater percentage of respondents from this sample working in the ‘Education’ and ‘Research’ sectors (table 1; electronic supplementary material, table S1). In reference to the period prior to the pandemic, respondents from both samples were similarly likely to select ‘Social media’, but in the period of the pandemic social media respondents were significantly more likely to select this option than their online panel counterparts (electronic supplementary material, table S5; chi-squared test: p-value<0.001).

Most participants stated that they had an ‘About right’ level of information of modelling during the pandemic with respondents from both samples being similarly likely to select this response (51% and 60% of the online panel and social media respondents, respectively; chi-squared test: $\chi_{df=1}^{2}=4.76;\;n=706;\;p\text{-value}=0.029$). Some of those selecting the option having an ‘About right’ amount of information also selected that they were unaware of modelling, demonstrating that not all have interest in this topic, in both samples (electronic supplementary material, figure S3; table S7).

### 3.3. Reliability of mathematical transmission modelling in informing policy

Online panel and social media respondents reported similar mean reliability scores of 6.3 (standard deviation (s.d.) 1.8) and 6.4 (s.d. 1.9), respectively, in reference to the pre-pandemic period (Mann–Whitney U test: p-value=0.493; electronic supplementary material, figure S4). During the pandemic, mean reliability scores rose significantly to 6.9 (s.d. 2.0) among online panel respondents (Wilcoxon signed rank test: p-value <0.001), but rose similarly among the social media respondents (mean 6.7 (s.d. 2.3); although this increase was not significant, Wilcoxon signed rank test: p-value=0.204, largely due to the more limited sample size [n=202 for social media compared with n=504 in the online panel]). There was no evidence to suggest significant differences between mean reliability scores across the two samples during the pandemic (Mann–Whitney U test: p-value=0.613).

**Figure 3.**
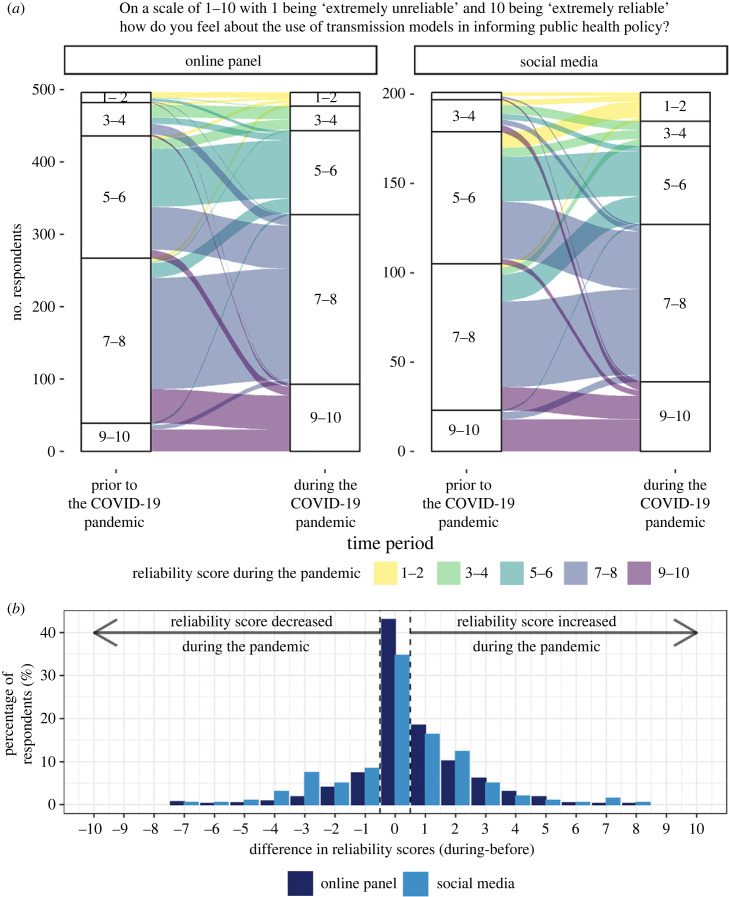
Reliability of using transmission modelling to inform public health policy. (*a*) Responses to the question ‘On a scale of 1–10 with 1 being ‘extremely unreliable’ and 10 being ‘extremely reliable’ how do you feel about the use of transmission modelling in informing public health policy?’ both ‘Prior to’ (reported retrospectively) and ‘During’ the COVID-19 pandemic, for both the online panel and social media samples. Responses were categorized into groups of 2 (from 1–2) to (9–10) and observations corresponding to a respondent which did not answer the question for at least one time point were removed from the figure to aid visual presentation (*n* = 8 online panel; *n* = 1 social media). Due to very few social media respondents reporting a reliability score in the category ‘1–2’, this option is unlabelled for the period ‘Prior to’ the pandemic but appears as the first option here as in three other response pillars. Underlying data are presented in table 1. (*b*) Distribution of the within-respondent differences in reliability scores from during the pandemic compared with prior for both the online panel and social media samples. Difference in scores is defined as the reliability score during the pandemic minus reliability score prior to the pandemic.

A majority of respondents changed their reliability score in reference to different time periods, with respondents from each sample being similarly likely to do so (figure 3; electronic supplementary material, table S8; online panel 55%; social media 65%; chi-squared test: $\chi_{df=1}^{2}=4.96;$ n=706; p-value=0.026). Respondents were similarly more likely to perceive greater reliability during the pandemic compared with before, regardless of sample (40% and 39% for online panel and social media, respectively; $\chi_{df=1}^{2}=0.04;\;\;n=706;\;p\text{-value}=0.840$). However, social media respondents were around 70% more likely to have a decreased feeling of reliability compared with their online panel counterparts (26% and 15% for social media and online panel, respectively; $\chi_{df=1}^{2}=10.34;\;n=706;$ p-value=0.001).

During the COVID-19 pandemic, being male was significantly associated with lower reliability scores compared with females within the social media sample only (electronic supplementary material, table S9; chi-squared test: p-value=0.001). Awareness was not associated with reliability score within this sample during the pandemic (p-value=0.817). By contrast, within the online panel in reference to both time points, increased awareness of the use of modelling in informing policy was associated with a significant increase in feeling of reliability rather than the demographic variables (electronic supplementary material, table S10; online panel and pre-pandemic: p-value<0.001; online panel and during: p-value<0.001).

### 3.4. Responsibility of communicating mathematical transmission modelling to the public

Almost all respondents believed that governments have the responsibility of ensuring that the public are informed about the use of modelling in informing policy decisions (92% of online panel respondents; 91% of social media respondents; table 1; electronic supplementary material, figure S10; chi-squared test: $\chi_{df=1}^{2}=0.10;\;n=706;\;\;p\text{-value}=0.756$). Social media respondents were significantly more likely to select those who ‘develop’ or ‘use’ transmission models, ‘The media’ and ‘Myself, as a member of the public’ as having the responsibility than online panel respondents (electronic supplementary material, table S13). Except for ‘Myself, as a member of the public’, the three aforementioned responses were similarly popular within each sample (electronic supplementary material, table S13).

### 3.5. Trust in government advice

**Figure 4.**
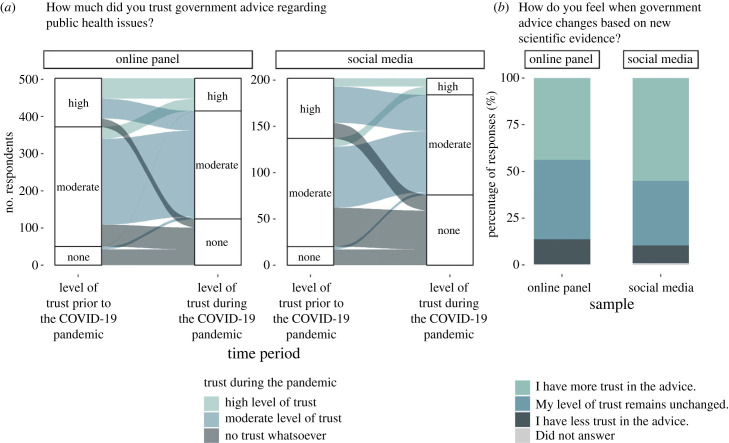
Trust in government scientific advice. (*a*) The percentage of observations from both the online panel and social media samples according to time period and answer to the question ‘How much did you trust government advice regarding public health issues?’ both ‘Prior to’ (reported retrospectively) and ‘During’ the COVID-19 pandemic. Observations corresponding to respondents who did not answer the question for at least one time point were removed from the figure to aid visual presentation (*n* = 1 respondent from the online panel). Underlying data are presented in table 1. (*b*) Answers to the question ‘How do you feel when government advice changes based on new scientific evidence?’ for both the online panel and social media samples. Underlying data are presented in table 1.

Most respondents reported having ‘moderate’ trust in the government regarding public health issues, regardless of sample or time period (table 1; figure 4*a*). Particularly in reference to the pre-pandemic period, many respondents stated their belief that the government had the best interest of the public at heart, with one stating ‘I didn't have [a] reason not to’ (online panel). Within both samples, respondents noted that they had a high level of trust in scientific evidence rather than the government: ‘I trusted government advice because this is provided by scientific advisors' (online panel) and ‘I trusted the scientist[s] not the politicians’ (social media), acknowledging that decisions taken by government can be influenced by more than just science: ‘It can be difficult to disentangle the science from politics' (social media). The perception of the government not trusting scientific advice was frequently mentioned as a reason for a lower level of trust, for example: ‘They didn't always listen to scientists, which deteriorated my trust’ (online panel). These views were reflected in the reliability scores with a higher level of trust in government advice being associated with higher median reliability scores for both samples. However, there was substantially greater variance in reliability scores among those with lower trust levels. (electronic supplementary material, figure S12; table S14).

Most social media respondents reported a different level of trust in government public health advice regarding the period of the pandemic compared with the pre-pandemic period (54%), significantly more than the minority of online panel respondents also doing so (35%) (chi-squared test: $\chi_{df=1}^{2}=22.51;\;n=706;\;p\text{-value}<0.001$) (electronic supplementary material, table S15). In particular, the percentage of respondents selecting having ‘No trust whatsoever’ rose significantly during the pandemic from 10% (both samples) to 25% and 38% for online panel and social media respondents, respectively (Wilcoxon signed rank test: Online panel: p-value<0.001; Social media: p-value<0.001). During the pandemic, 26% of social media respondents and 18% of online panel respondents went from a ‘High level of trust’ to ‘No trust whatsoever’ (chi-squared test: $\chi_{df=1}^{2}=1.48;\;n=706;\;p\text{-value}=0.223$). Reasons given for this included: ‘Felt that they [the government] were more driven by political and economic priorities rather than public health’ (online panel) and ‘Not convinced they followed the science on every occasion as they stated they were doing’ (social media). Separately, another respondent noted: ‘My trust has reduced but not yet to the position of no trust’ (online panel). Together, this demonstrates an erosion of trust among respondents, but substantially more so among the social media sample.

One respondent who went from ‘High’ to ‘No’ trust gave the following reason for their opinion: ‘Government kept changing the advice given during the pandemic’ (online panel). However, when asked directly about trust in relation to the government changing their advice based on scientific evidence, a minority of respondents from both samples stated consequently having ‘Less trust’ (table 1; figure 4*b*; 13% and 10% for online panel and social media, respectively; chi-squared test: $\chi_{df=1}^{2}=1.69;\;n=706;\;p\text{-value}=0.194$). Among those stating they find changing advice ‘Less trustworthy’, respondents noted: ‘It makes me feel as though the government make up things to give the public false hope until there is scientific research available to support’ (online panel) and ‘Gets too confusing [with] mixed messages' (social media). Others among the online panel sample suggested that it was the rapid pace at which advice changed which influenced their level of trust: ‘the advice changed so frequently it was hard to know what to believe was the right thing to do’.

Social media respondents (56%) were (borderline) ‘More trusting’ of changing advice based on new evidence than their online panel counterparts (44%) (chi-squared test: $\chi_{df=1}^{2}=6.70;\;n=706;\;p\text{-value}=0.010$), although this was still the most common response among both samples. Respondents noted: ‘It shows a willingness to address a changing situation’ (online panel) and ‘During a pandemic there will be need to amend advice as more data becomes available and more [is] known about the virus' (social media). Across both samples, it was clear that respondents placed a high value on understanding the source of the new information (‘More trust as long as sources are quoted’ (online panel)) and the method in which it was explained to the public (‘I understand that evidence as it gathered can lead to new understanding and therefore improved modelling. However, it can be dependent on how well it is explained and communicated’ (social media)).

## 4. Discussion

This study was undertaken to complement the findings of our previously published work on the relationship between modelling and policy [2] by providing insights into the UK public's awareness and opinion of transmission modelling in informing policy. As such, novel data were gathered under two different sampling mechanisms, an online panel (‘representative’ sample) and social media (‘non-probability’ sample) and in respect of two time periods, ‘Prior to’ and ‘During’ the COVID-19 pandemic.

Almost all respondents reported being increasingly aware of the use of modelling in informing policy during the pandemic, with significantly higher levels of awareness among social media respondents than online panel respondents. Awareness generally stemmed from the news media and social media during the pandemic. The mean reliability score increased during the pandemic compared with the (retrospective) pre-pandemic period under both samples, with awareness being positively associated with reliability only within the online panel. Trust in government public health advice remained high across samples and time periods overall but was lower in the period of the pandemic compared with the pre-pandemic period. The decay in trust was notably greater among social media respondents. Many respondents from both samples explicitly made the distinction that their trust was reserved for ‘scientists’ and not ‘politicians’. Almost all respondents, regardless of sample, believed governments have responsibility for the communication of modelling to the public.

Our results are not, nor claim to be, a definitive representation of all public opinion on this topic. Instead, we have focused primarily on the differences in the responses between our two samples. Specifically, our results validate our *a priori* expectation of a skewed response among social media respondents thus providing an important reminder of the potentially biased conclusions that could be drawn from non-representative samples [21]. Throughout, it is important to recall that social media respondents were likely to have accessed the survey directly from a Twitter account they follow related to public health or mathematical modelling, thus demonstrating an active interest in and/or familiarity with the subject matter (electronic supplementary material, table S1). By contrast, online panel respondents saw this survey without any context. Furthermore, social media respondents were significantly more likely to work in ‘Research’ or ‘Education’ than online panel respondents, which may account for a substantial amount of the differences observed in the responses received, given that modelling for public health policy was primarily undertaken by academics [5].

While the general sentiment of the responses was consistent across samples, often social media respondents were more likely to have stronger or polarized views. Examples of this include trust in the government decreasing within both samples but at a greater level for social media respondents compared with the online panel, and almost all social media respondents stating awareness of the use of modelling in informing policy compared with three-quarters of online panel respondents. In general, age and gender were only found to be significantly associated with or approaching significant association for responses among the social media sample. Nonetheless, caution is still needed when interpreting the results from the online panel as the suitability of this sample as a representation of the UK population is unclear [18]. However, 87% of online panel respondents (aged over 18 years old) stated having had at least one dose of a vaccine for SARS-CoV-2 compared with 76.9% of the population over 12 years old in the UK on 9 July 2021 (when online panel survey responses were gathered) [22]. As vaccination of those aged under 18 years was not recommended in the UK until 15 July 2021 [23], it is reasonable to consider 76.9% a lower bound of vaccination coverage of the UK adult population thus suggesting reasonable representativeness of the online panel with regards to this variable.

Given the role that modelling has played in the pandemic response in the UK [2,3,5,6], and the corresponding coverage in the media [24–27], it is unsurprising that respondents reported an increased awareness of the use of modelling as a tool to inform public health policy. However, it is perhaps unexpected that this translated to most participants in both samples. The popularity of the news media as a means of awareness underlines the important role that journalists play as science communicators, and emphasizes the value of organizations such as the Science Media Centre [28]. A number of the experts interviewed as part of our aforementioned study praised the commitment that many journalists showed to clear and accurate communication of modelling studies [2]. Professor Sir Chris Whitty also spoke about communicating modelling to the public during the pandemic during his testimony to the UK COVID-19 Inquiry in November 2023, noting that this has to be done with ‘great care’ and in a way that is different to presenting raw data [29]. The rise in popularity of social media as a means for being aware of modelling is unsurprising among social media respondents, but the significant fourfold increase among online panel users in the period of the pandemic was particularly notable. Akin to the case of the news media, this finding further demonstrates the role that social media can play in science communication, particularly in the COVID-19 pandemic and, consequently, the importance of ensuring that studies are properly represented to users of this platform by those sharing them.

One of the major findings of the authors' initial science-policy paper was the importance of distinguishing between scientists and decision-makers, with only the latter being directly accountable to the public [2]. Many respondents, from both samples, noted this distinction explicitly when commenting on the question regarding trust in government public health policy, and within both samples during the pandemic respondents were significantly more likely to state that modellers work within ‘Academia’ rather than ‘Government’ (see the electronic supplementary material). While this suggests a reasonable understanding of the difference between these two roles, further and continued emphasis of the difference in roles may be warranted ahead of future emergencies, public health-related or otherwise, as ‘Government’ became a significantly more popular response during the pandemic compared with the pre-pandemic period among the online panel. Nearly all respondents from both samples indicated that the government have the responsibility of ensuring that the public are informed of scientific evidence underpinning their decisions. This remains compatible with another key finding of our initial study that science must be communicated by scientists [2], for example by having scientists present at government-organized press conferences.

There will often be tensions between scientific advice and decision-making. This was particularly evident during the COVID-19 pandemic when our understanding of SARS-CoV-2 was rapidly evolving as new data became available, with public health guidance changing almost equally rapidly to reflect new findings. This was highlighted by science communication experts in our previous study as a key challenge of communicating with the public and was encapsulated well by a social media respondent: ‘Politicians don't like u-turns while scientists are keen to constantly evolve thinking and theories. These outlooks are not particular[ly] compatible when it comes to communicating subtle changes in public health messaging’. However, most respondents from both samples noted that changing advice either did not change or increase their trust in said advice, although this was significantly more common among social media respondents. Our results indicate a key factor of trust is transparency of evidence, also highlighted in our initial study [2], rather than rapidly changing advice.

The fact that respondents reported ‘No awareness’ of modelling in informing policy and that for some (online panel n=10; social media n=1) this was an ‘About right’ level of knowledge provides an important reminder that this topic is, understandably, not of interest to all. Furthermore, while awareness was associated with increased feelings of reliability of modelling, the fact that some ‘Aware’ respondents selected a reliability score in the lower half of the scale during the pandemic highlights the complexities of this relationship. Nonetheless, some respondents seemed to show a genuine interest and appreciation of modelling: ‘I have found a new respect for those who do modelling, to be honest I find it fascinating’ (online panel).

It is important to note the limitations of this study. All results rely on the opinions and experiences of our participants. Although we distributed the survey via two platforms, as discussed we are unable to ascertain the representativeness of the online panel in terms of the UK population, as is cautioned by Prolific Academic [18]. Furthermore, although respondents were asked questions in relation to the period ‘Prior to’ and ‘During’ the COVID-19 pandemic separately, sampling only took place during the latter period. Therefore, responses for the pre-pandemic period may have been influenced by recall bias and should be interpreted with caution. Due to the nature of the sampling platforms, all online panel responses were obtained in a much shorter period (days) than social media responses (weeks). Around this time, all public health restrictions in England were lifted after a one-month delay [30], which could have influenced participants' responses. We also note that the limited sample sizes obtained under the two sampling mechanisms (*n* = 504 for the online panel and n=202 for social media) necessitates caution when interpreting differences between the two samples. Finally, although respondents were given multiple opportunities to add open-ended comments, multiple-choice answers may lose some of the nuance of participants’ opinions.

As noted by one respondent: ‘I think we have all learned something about science and how it impacts on our daily lives throughout the pandemic’. Ultimately, the public are key stakeholders of public health policy, and if mathematical transmission models are used to inform important decisions, then there is a responsibility to assess people's understanding of and feelings towards them. We hope that the findings of this study will be useful in informing science communication strategies in future emergencies. For example, by being more transparent about the evidence underpinning the implementation of or rapid change to policy, or by better using the news media and/or social media as means of communicating with the public.

## Data Availability

In line with the ethics approval obtained, raw research data is accessible to the Medical Sciences Interdivisional Research Ethics Committee at the University of Oxford, upon their request. Anonymized summary data are provided in table 1. Code for R analyses are provided from the Zenodo repository: http://dx.doi.org/10.5281/zenodo.10201375 [31]. The multiple-choice data are provided in electronic supplementary material [32].
